# Flipping the tumor from outward to inward: simplifying and accelerating endoscopic full-thickness resection

**DOI:** 10.1055/a-2760-9980

**Published:** 2026-01-15

**Authors:** Youzhu Lu, Yiting Huang, Qide Zhang

**Affiliations:** 1 688090 Digestive Endoscopy Center, Affiliated Hospital of Nanjing University of Chinese Medicine, Jiangsu Province Hospital of Chinese Medicine, Nanjing, China; 2 66478 Nanjing University of Chinese Medicine, Nanjing, China

For gastric submucosal tumors (SMTs) originating from the muscularis propria (MP), particularly those with extraluminal growth, endoscopic full-thickness resection (EFR) is a reliable endoscopic technique 1 2 that can achieve en bloc resection during surgery. Nonetheless, it remains a challenging and complex procedure due to the risk of the tumor falling into the abdominal cavity and the potential for severe bleeding from the thick blood vessels.

**Fig. 1:**
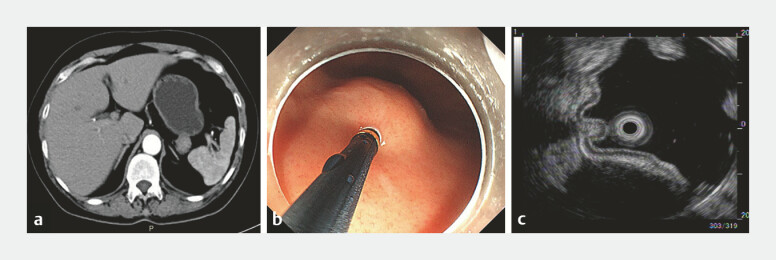
**a** Contrast-enhanced abdominal computed tomography (CT) showed gastric fundus posterior wall mass. **b** White light endoscopy revealed a SMT at the gastric fundus. **c** EUS revealed submucosal tumor of the gastric fundus (extraluminal growth-type). EUS, endoscopic ultrasound; SMT, submucosal tumor.

**Fig. 2:**
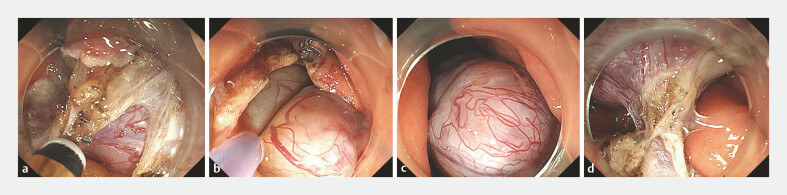
**a** The tumor was exposed. **b** The tumor was grasped using a snare. **c** The tumor was retracted into the gastric cavity. **d** Excellent visualization achieved.

**Fig. 3:**
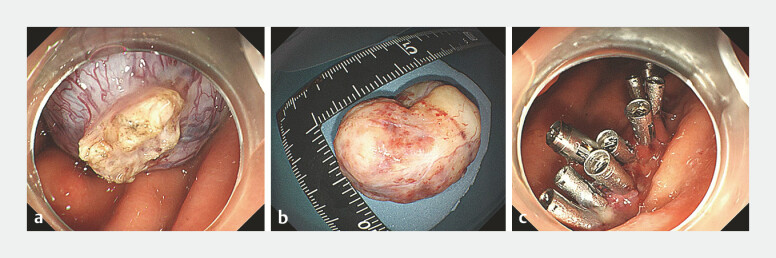
**a** The tumor is completely detached. **b** The tumor successfully excised and extracted. **c** The sutured wound.

A 54-year-old woman presented with a 5-month history of abdominal discomfort. Contrast-enhanced abdominal computed tomography revealed an extraluminal iso-echoic tumor ( Fig. 1 **a** ). White light imaging disclosed a SMT at the gastric fundus ( Fig. 1 **b** ), and endoscopic ultrasonography indicated a homogeneous hypoechoic extraluminal growth-type tumor originating from the MP ( Fig. 1 **c** ). Following the standard EFR procedure ( Fig. 2 **a** ), we incised the gastric wall and inspected a portion of the tumor. Subsequently, we used a snare to capture the tumor and reposition it into the gastric cavity ( Fig. 2 **b, c** ). The tumor's root from the gastric wall was visualized and dissected smoothly ( Fig. 2 **d** ). After the tumor was completely detached, the snare was used once more to extract it from the body. The tumor measured 30 mm × 35 mm in size, and subsequently, the wound was sutured successfully with clips ( Video 1 , Fig. 3 **a–c** ). The entire operation took about 15 minutes, with no intra- or post-operative bleeding, and there were no significant abdominal gas complications. The patient was orally consuming liquid food on day 2 post-operation and was discharged uneventfully on day 4. The pathological results indicated a low-risk gastrointestinal stromal tumor.

Flipping the tumor from outward to inward: simplifying and accelerating endoscopic full-thickness resection.Video 1

By drawing the tumor from outside into the lumen during the EFR, the boundary of the tumor, along with the thick blood vessels of the serosal layer and extraluminal vessels, could be clearly observed.

Endoscopy_UCTN_Code_TTT_1AO_2AG_3AF
